# A randomized double-blind active-controlled clinical trial on the efficacy of topical basil (*Ocimum basilicum*) oil in knee osteoarthritis

**DOI:** 10.3389/fphar.2024.1377527

**Published:** 2024-05-06

**Authors:** Alireza Askari, Fatemeh Sadat Hasheminasab, Omid Sadeghpour, Mohammad Mehdi Naghizadehd, Seyed Ali Ravansalar, Aida Iraji, Mohammad Hashem Hashempur

**Affiliations:** ^1^ Bone and Joint Reconstruction Research Center, Shafa Orthopedic Hospital, Iran University of Medical Sciences, Tehran, Iran; ^2^ Pharmacology Research Center, Zahedan University of Medical Sciences, Zahedan, Iran; ^3^ Department of Genetic, School of Medicine, Zahedan University of Medical Sciences, Zahedan, Iran; ^4^ Department of Traditional Pharmacy, School of Persian Medicine, Iran University of Medical Sciences, Tehran, Iran; ^5^ Non-Communicable Diseases Research Center, Fasa University of Medical Sciences, Fasa, Iran; ^6^ Student Research Committee, Shiraz University of Medical Sciences, Shiraz, Iran; ^7^ Research Center for Traditional Medicine and History of Medicine, Department of Persian Medicine, School of Medicine, Shiraz University of Medical Sciences, Shiraz, Iran

**Keywords:** osteoarthritis, herbal medicine, traditional Persian medicine, basil, *Ocimum basilicum*

## Abstract

**Background:**

Basil is a widely used herb in Persian medicine and is gaining recognition as a functional food worldwide.

**Aim of the study:**

This trial aimed to assess the effectiveness of a traditional formulation of basil oil in comparison with diclofenac gel in treating knee osteoarthritis, considering its established anti-inflammatory, anti-nociceptive, and anti-oxidative properties.

**Materials and methods:**

One hundred eligible patients were equally randomized to the traditional basil oil (containing sesame oil) and diclofenac gel groups. They used their respective topical treatments thrice daily for 4 weeks. Various measurements were taken at the beginning of the study, 2, and 4 weeks after starting the intervention, including the 8-m walk test, knee pain (based on visual analog scale), flexion angle of the knee joint, analgesic consumption, and the Western Ontario and McMaster Universities Osteoarthritis Index (WOMAC) questionnaire.

**Results:**

No significant differences were observed between the basil oil and diclofenac gel groups in any of the measured outcomes. However, significant improvements were noted within each group for most variables.

**Conclusion:**

Topical application of the traditional formulation of basil oil appears to improve clinical symptoms and certain functional indicators of knee osteoarthritis to a similar extent as diclofenac gel. This suggests that basil oil could be considered an effective management option for this condition.

**Clinical Trial Registration:** https://irct.behdasht.gov.ir/, identifier IRCT2017081711341N7.

## 1 Introduction

Osteoarthritis (OA) is known as a common disease among all different musculoskeletal disorders (Pereira et al., 2011; Hallberg et al., 2023). It significantly impacts various aspects of quality of life, including reduced mobility, dexterity, and function; persistent pain; and decreased mental wellbeing. Therefore, OA can cause social, economic, and functional dependence for patients. Additionally, insufficient physical activity increases the risk of other non-communicable diseases (Salaffi et al., 2005; Briggs et al., 2016).

The OA prevalence enhances with age, with around 75% of individuals aged 65 years and older experiencing OA; also, nearly 40% of adults over 70 years of age suffer knee OA (Brooks, 2002; Cook et al., 2007). Other factors associated with a raised OA risk include female gender (Sowers, 2001), low socioeconomic status (Thumboo et al., 2002), high body mass index (Lee and Kean, 2012), and race (Golightly and Dominick, 2005). Recent reports indicate that the prevalence of knee OA is higher than hip OA (Picavet and Hazes, 2003; Andrianakos et al., 2006; Pereira et al., 2011).

OA is an incurable condition, and its management primarily focuses on symptom relief. Traditional treatment approaches for knee OA involve addressing disability and managing pain. Additionally, lifestyle modifications and surgery may be recommended in severe cases to slow down disease progression (Bennell et al., 2012). The commonly recommended analgesics for managing this disorder are Non-steroidal anti-inflammatory drugs (NSAIDs). These drugs may induce several adverse effects on the cardiovascular and gastrointestinal systems, particularly in older patients (Harirforoosh et al., 2014; VanAtta and Perry, 2023).

Recent studies have shown that a significant number of OA patients turn to complementary and alternative medicine for relief. One study reported that approximately 84% of individuals with musculoskeletal symptoms utilized alternative therapies (Cronan et al., 1989). Another study found that 47% of subjects with OA used an alternative care; alternative medications were used by 17.2% of them (Ramsey et al., 2001). Patients are drawn to these treatment methods due to factors such as availability, natural origin, safety, or unknown side effects (Bodeker and Ong, 2005). The widespread use of complementary and alternative medicine, particularly medicinal plants, highlights the need for thorough investigation and evaluation of their efficacy.

One of the herbal remedies recommended for treating joint disorders is *Ocimum basilicum* L., known as basil (Machado et al., 2019). Basil is a widely recognized herb globally. Avicenna (10th and 11th centuries), Rhazes (ninth and 10th centuries), and Salehi Heravi (18th century) have mentioned the usefulness of basil in reducing inflammation, and alleviating some gastrointestinal disorders such as bloating and hemorrhoid. Moreover, the traditional formulation of basil oil (including sesame oil as its vehicle) has been specifically recommended for relieving joint pain (Salehi Heravi, 1989 (in Persian), Rhazes, 2015; Avicenna, 1999). Recent research has demonstrated its excellent antioxidant properties (Amrani et al., 2006), attributed to its high polyphenolic and flavonoid content (Sestili et al., 2018). Various studies have confirmed the anti-nociceptive (Venâncio et al., 2011; Hosseinzadeh et al., 2012) and anti-inflammatory (Aye et al., 2019; Eftekhar et al., 2019; Beheshti et al., 2023) properties of this medicinal plant.

Several recent trials have explored the various properties of basil. In a triple-blind randomized clinical trial, the impact of the oral capsule containing basil leaf extract on severity of insomnia and sleep quality in menopausal women was examined. The findings of this study indicated that the consumption of basil capsules resulted in improved sleep quality and reduced insomnia in menopausal women (Karimi et al., 2023). Another trial investigated the effects of the oral capsule of basil leaf extract on depression in menopausal women, revealing a decrease in depression scores among those who consumed the basil leaf extract capsules (Karimi et al., 2021). Additionally, a pilot clinical trial examined the potential of basil leaves as an adjunct therapy for stage 1 and 2 hypertension. Participants were randomly assigned to either the control group or the basil group, with the control group receiving their prescribed antihypertensive medication along with a placebo, and the basil group receiving their antihypertensive medication along with basil capsules. Blood pressure measurements were taken at baseline, 1 week, and 2 weeks after the start of the drug administration. The results demonstrated significant differences in systolic and diastolic blood pressure levels in the basil group compared to the control group over the course of the study period (Ratta et al., 2021).

Based on the aforementioned effects of the traditional formulation of basil oil in traditional Persian medicine, supported by recent scientific research, this trial was performed to examine the efficacy and safety of topical basil oil in ameliorating knee OA.

## 2 Materials and methods

### 2.1 Ethics and informed consent

The local Medical Ethics Committee of Fasa University of Medical Sciences (FUMS) approved the protocol of this clinical trial (Code: IR. FUMS.REC.1394.39). Additionally, the study protocol was registered on the Iranian Registry of Clinical Trials website (IRCT2017081711341N7). This investigation was carried out in accordance with the guidelines of the Declaration of Helsinki (1989 revision) and the IAHAIO Tokyo Declaration. Patients were informed about the trial procedure, and if there was consent to participate in the study, they signed the relevant form.

### 2.2 Patients recruitment

Patients diagnosed with OA considering the criteria of the American College of Rheumatology (Altman et al., 1986) and Kellgren-Lawrence Grading Scale (Kellgren and Lawrence, 1957) (grade I to III), who were between 30 and 70 years old., and had experienced knee pain for 3 months or more were included in this research. Exclusion criteria were pregnancy, lactation, obesity (BMI≥35 kg/m^2), and secondary OA (e.g., metabolic arthritis, infected arthritis, gout, pseudo gout, and traumatic arthritis). Individuals with a past medical history of joint replacement surgery, inflammatory bowel disease, peptic ulcer, knee skin lesions, or drug abuse were also excluded. Additionally, patients who had used topical or oral analgesic medication within 3 days prior to the study, used glucosamine or chondroitin sulfate, received corticosteroids or hyaluronic acid injections in their knees during the last 3 months, consumed oral or topical corticosteroids within 14 days before enrollment, or underwent any physical modalities (such as transcutaneous electrical nerve stimulator, acupuncture, and physiotherapy) within 2 weeks prior to evaluation were not eligible to participate.

### 2.3 Preparation and standardization of the study drug

To prepare the traditional formulation of basil oil, the required herbs including fresh basil leaves and sesame seeds were purchased from an herbal shop. The samples of them were transferred to the Department of Pharmacognosy, Faculty of Pharmacy, Kerman University of Medical Sciences and voucher codes was assigned for basil (KF1368) and sesame (KF1674).

Basil leaves were pounded and pressed them to extract the juice. The juice extracted from basil leaves was combined with cold press sesame (*Sesamum indicum* L.) oil in a weight proportion of 2:1. It was heated gently to evaporate the water content, leaving behind the oil.

The basil oil fatty acid compound was analyzed via gas chromatography (GC)-flame ionization detector (FID) after methyl ester derivatization. Methyl ester derivatives of fatty acids were obtained based on the guideline provided in ISO 12966-2:2017 (https://www.iso.org/standard/72142.html). To identify the fatty acids, we compared their retention times with those of standard compounds injected into the GC–FID system immediately after sample injection with the same protocol.

### 2.4 Study design

This prospective randomized double-arm, double-blind active-controlled clinical trial was carried out to compare the efficacy of basil oil *versus* diclofenac sodium gel (%1) in individuals suffering from knee OA. The allocation ratio was considered 1:1, and the study method remained unchanged after the approval of the proposal.

### 2.5 Study setting

This study was carried out in the orthopedic clinic affiliated with FUMS, Fasa. Patients were initially evaluated by an orthopedic specialist and then introduced to the researchers upon clinical diagnosis of knee OA. Eligible cases were invited to take part in the study. Patient recruitment occurred between September 2018 and April 2019.

### 2.6 Randomization and blinding

All patients who were diagnosed with osteoporosis based on the diagnosis of an orthopedic specialist and visited the aforementioned clinic underwent simple sampling. Given the inclusion and exclusion criteria, 100 knee OA subjects were selected, randomized and allocated to either the control or intervention groups. A biostatistician used the blocked randomization method (non-stratified, with four participants in each block) by Microsoft Excel^®^ software to generate the randomization list for this trial. To ensure allocation concealment, opaque and sealed packets containing numbers “1” and “2” were prepared. A clinic secretary allocated the participants to the study groups by sequentially selecting packets with the corresponding numbers.

Both basil oil and diclofenac gel were prepared in identical containers with similar characteristics. The contents of the drugs were undisclosed to the patients, physicians, and outcome assessors. A third person, who had no role in the study, gave the drugs to the participants.

### 2.7 Intervention and compliance

At first, the researchers obtained the written informed consent from the participants; then, they were randomized to either the diclofenac sodium gel 1% (control) or basil oil (intervention) groups. They received basil oil or diclofenac gel (Sobhan Darou) to apply on the knee (1.5 cc or one fingertip unit, respectively) thrice daily over a period of 4 weeks. All participants were provided with capsules containing 100 mg of celecoxib (Tehran Darou); to the maximum allowed daily dose of celecoxib for pain relief was 200 mg.

In our clinical trial, compliance was assessed through a combination of patient self-reporting and monitoring during follow-up visits. Patients were provided with a daily diary to record their usage. During follow-up visits, patients were asked about their adherence to the treatment regimen, and any deviations or issues with compliance were documented. Additionally, any unused product was collected and accounted for at each visit to ensure that patients were not using the product more frequently than recommended. This approach allowed us to monitor and track patient compliance throughout the study duration. Additionally, participants were advised to maintain their usual lifestyle and dietary habits to minimize potential fluctuations in weight that could impact the study outcomes.

### 2.8 Outcome measures

The primary outcome of this investigation was measured using a validated questionnaire known as the Western Ontario and McMaster Universities Osteoarthritis Index (WOMAC) score (Bellamy et al., 1988).

The secondary outcomes were assessed as follows.

- Measurement of knee stiffness and physical function using the WOMAC questionnaire
- Knee pain assessment using the WOMAC questionnaire, and Visual Analogue Scale (VAS)
- Estimation of the time required to walk on a standard 8-m flat surface
- Measurement of the flexion angle of the knee through a standard goniometer
- Quantification of the amount of celecoxib used

Additionally, adverse events were elicited and assessed through a structured approach, during follow-up visits. The monitoring process involved the use of a checklist comprising a series of inquiries pertaining to different physiological systems, encompassing the skin, cardiovascular, nervous, respiratory, and gastrointestinal systems.

### 2.9 Sample size

Based on a study by Lei et al. (Lei et al., 2017), knee problems measured by the WOMAC scale decreased from 43.7 ± 12.7 to 22.5 ± 10.3 over 6 months. Given a power of 90% and a 5% error rate, a minimum of 43 individuals in each group were needed to obtain an 8-unit reduction in the WOMAC index. Taking into account the possibility of participant dropouts, we initiated the trial with a 20% larger sample size of 50 individuals in each group.

$$n=\frac{s_{1}^{2}+s_{2}^{2}}{{\left(\bar{x_{1}}-\bar{x_{2}}\right)}^{2}}{\left(Z_{1-\frac{\alpha }{2}}+Z_{1-\beta }\right)}^{2}$$

s1 = 12.7, s2 = 10.3, α = 0.05, Z1-α/2 = 1.96, β = 0.1, Z1-β = 1.28.

### 2.10 Statistical analysis

The mean and standard deviation (SD) were used to present the data. The chi-square test (sex, occupation) and *t*-test (age, BMI, disease duration) were applied to compare the baseline variable between the two groups.

Several factors were evaluated for each patient, including two WOMAC sub-scores (physical function and stiffness), walking speed, and analgesic consumption. The comparison of these factors at three different time points was conducted using Repeated Measurement ANOVA, followed by Bonferroni *post hoc* test within each group. The variables were compared between the study groups using t-tests at baseline and ANCOVA at 2 and 4 weeks. The latter analysis was adjusted for baseline measurements as a covariate. For variables assessed individually for each knee (such as knee flexion angle and VAS), as well as the total score of the WOMAC questionnaire and pain, a generalized estimate equation (GEE) model with an unstructured correlation matrix was employed for data analysis. GEE is an approach that accounts for correlated data without fully specifying the distribution of responses within each patient as a cluster (Hanley et al., 2003). The analysis focused on examining the variation of the mentioned variables separately for each group across three different evaluation times. In this analysis, the correlation between the three evaluation times and the two knees of each patient was taken into account. Another GEE model was used to compare the baseline characteristics between the study groups, treating the two knees as the correlated and the study group as the fixed factors. Two additional GEE models were employed to compare the study groups at 2 and 4 weeks after the intervention. In these models, the correlation between the knees was considered, with baseline measurements as the covariates and the study group as the fixed factor.

All of the statistical analyses were done using IBM SPSS 24 (IBM Corp. Released 2016. IBM SPSS Statistics for Windows, Version 24.0. Armonk, NY: IBM Corp.). For drawing the charts Microsoft Excel (Microsoft Co, Redmond, USA) was used. A *p*-value less than 0.05 was considered as the significant level.

## 3 Results

### 3.1 Basil oil analysis and standardization

Following the methylation of the basil oil, GC–FID was employed to determine and identify the oil fatty acids compounds. The identified fatty acids are presented in Table 1. The predominant fatty acid detected was linoleic acid (42.42%), followed by oleic acid (40.83%) and palmitic acid (11.98%).

**TABLE 1:** Type and amount of fatty acids found in basil oil.

Fatty acid	Amount (%)
Lauric acid	0.04
Myristic acid	0.05
Palmitic acid	11.98
Palmitoleic acid	0.07
Heptadecanoic acid	0.04
Stearic acid	3.00
Oleic acid	40.83
Linoleic acid	42.42
α-Linolenic acid	0.40
Arachidic acid	0.10
Docosanoic acid	0.05
Tetracosanoic acid	0.08
Others	0.94

### 3.2 Baseline data and study flow

**FIGURE 1:**
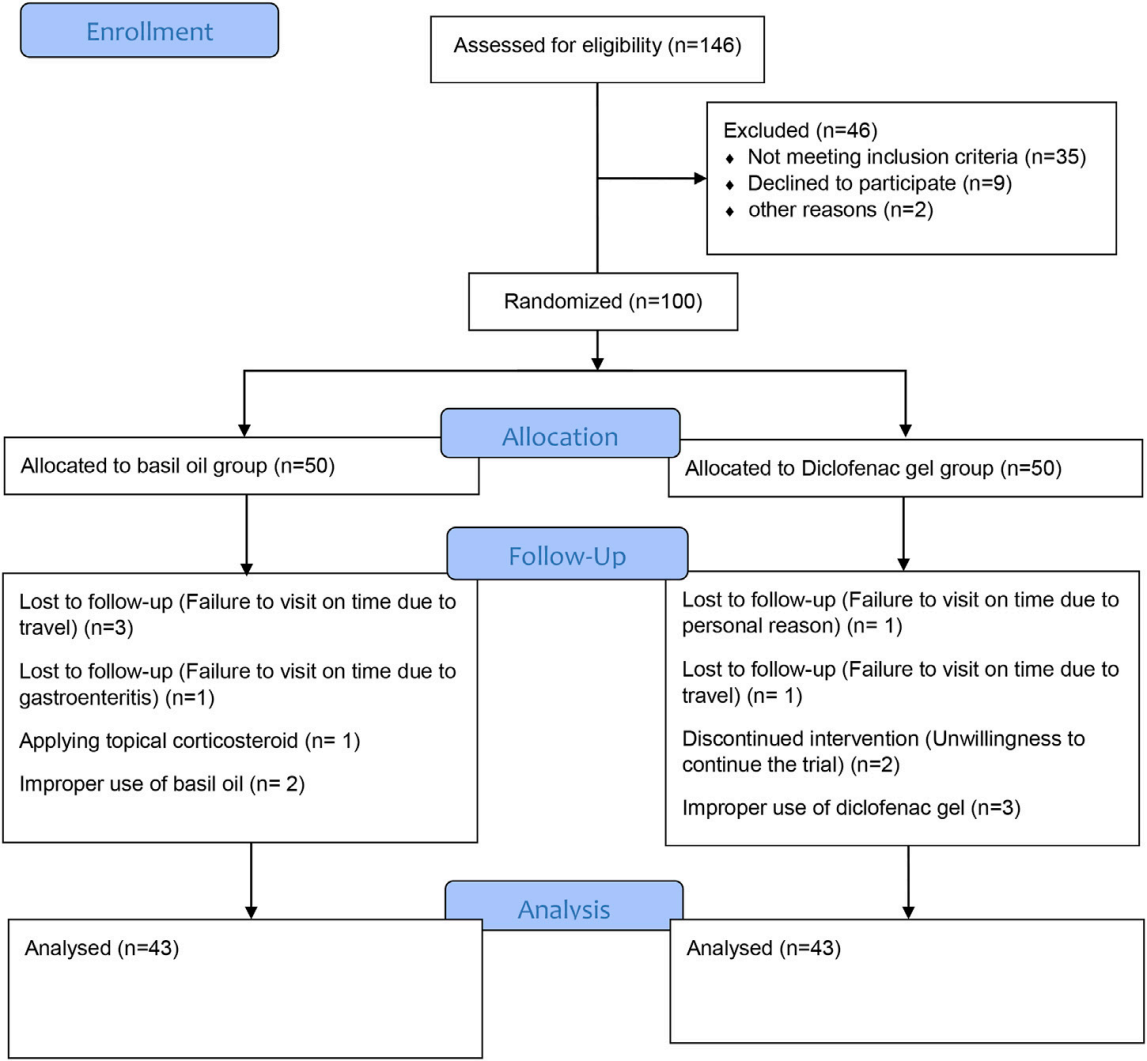
Trial CONSORT flow chart.

The eligibility of a total of 146 patients was evaluated from September 2018 to April 2019. After applying the inclusion criteria, we selected 100 eligible subjects for this trial and randomly allocated them to the intervention or control groups, with 50 cases in each group. Throughout the study, seven patients from the control group and seven cases from the intervention group were excluded because of such reasons as loss of follow-up, application of topical corticosteroids, or improper use of diclofenac gel or basil oil. Ultimately, 86 participants successfully completed the trial. The baseline demographic information is indicated in Table 2, which shows no significant differences in the measured clinical or demographic factors between the two groups at the beginning of the trial. Figure 1 displays the trial flowchart.

**TABLE 2:** Demographic characteristics of the patients participated in the study.

Demographic information	Basil oil (n = 43)	Diclofenac gel (n = 43)
Age (years), Mean ± SD	60.2 ± 9.1	57.2 ± 9.0
BMI^a^ (Kg/m2), Mean ± SD	28.0 ± 3.8	27.7 ± 4.5
Duration of disease (Month), Mean ± SD	83.0 ± 55.9	87.3 ± 68.2
Male/female (n)	6/37	8/35
Employed/Unemployed or Retired (n)	39/4	38/5

^a^ Body mass index.

### 3.3 Clinical outcomes

The participants in each group were followed for a duration of 4 weeks starting from the initiation of the intervention. The outcomes were assessed at beginning of the study, 2, and 4 weeks after starting the intervention. No statistically significant differences were seen between the intervention and control groups regarding pain severity based on the VAS scale (*p* values: 0.619 and 0.880), knee flexion angle (*p* values: 0.993 and 0.473), 8-m walking test (*p* values: 0.436 and 0.555), number of analgesic consumption (*p* values: 0.127 and 0.117), pain severity based on WOMAC questionnaire score (*p* values: 0.642 and 0.887), physical function based on WOMAC questionnaire score (*p* values: 0.744 and 0.239), knee stiffness (*p* values: 0.202 and 0.658), and total WOMAC scores (*p* values: 0.970 and 0.354) at the 2-week and 4-week intervals, respectively.

**FIGURE 2:**
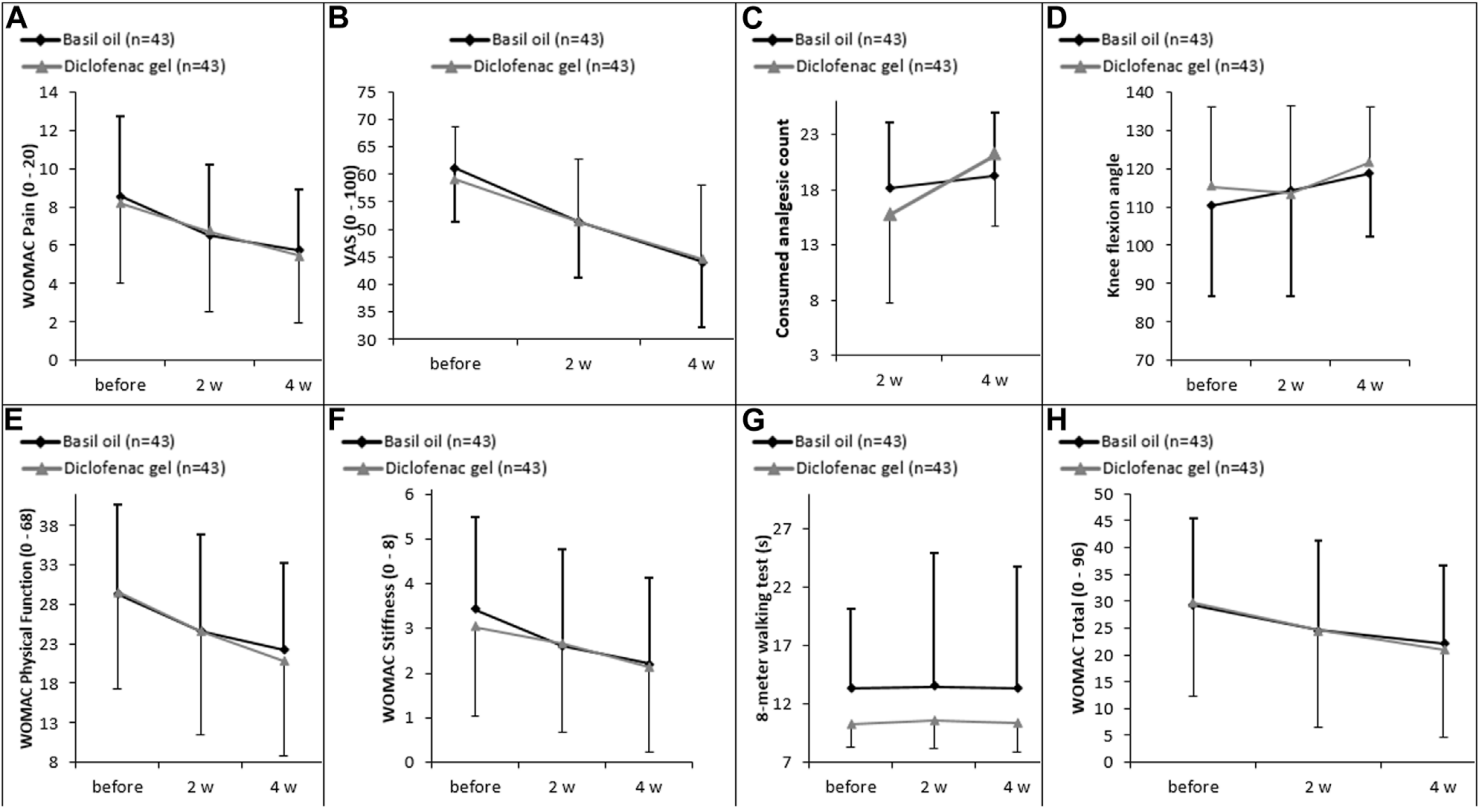
**(A)** Changes in intensity of pain measured by WOMAC scale, **(B)** changes in intensity of pain assessed by VAS scale, **(C)** the count of celecoxib used, **(D)** knee flexion angle, **(E)** physical function, **(F)** knee stiffness, **(G)** 8-m walking test, and **(H)** WOMAC score over the trial period between diclofenac gel and basil oil groups.

However, the analysis within each group showed significant differences in most variables, including pain severity based on the VAS scale or WOMAC questionnaire scores, physical function, knee stiffness, and total WOMAC scores when comparing baseline and the second week. Similarly, within-group analysis showed significant differences in the outcomes such as pain severity based on the VAS scale or WOMAC questionnaire scores, physical function, knee stiffness, total WOMAC scores, and knee flexion angle when comparing baseline and the fourth week (Figure 2; Table 3, 4).

**TABLE 3:** Comparison of outcomes (mean ± SD) between basil oil (intervention) and diclofenac gel (control) groups, and also before and after the intervention in each groups.

		Basil oil (n = 43)	Diclofenac gel (n = 43)	*p*-value^1^
**Visual analogue scale (VAS)**	Baseline	61.05 ± 9.67	59.07 ± 9.47	0.821
	2 weeks	51.28 ± 10.12	51.40 ± 11.46	0.619
	4 weeks	44.07 ± 11.87	44.53 ± 13.53	0.880
	P-value^a^	**<0.001**	**<0.001**	
	P-value^b^	**<0.001**	**<0.001**	
	P-value^c^	**<0.001**	**<0.001**	
**Knee flexion angle**	Baseline	110.41 ± 23.86	115.23 ± 20.74	0.304
	2 weeks	114.30 ± 27.65	113.49 ± 22.75	0.993
	4 weeks	118.72 ± 16.50	121.69 ± 14.21	0.473
	P-value^a^	0.087	0.922	
	P-value^b^	**0.006**	**0.027**	
	P-value^c^	0.507	**0.011**	
**8-m walking test**	Baseline	13.34 ± 6.81	10.30 ± 2.03	**0.006**
	2 weeks	13.49 ± 11.44	10.61 ± 2.45	0.436
	4 weeks	13.30 ± 10.47	10.39 ± 2.56	0.555
	P-value^a^	0.895	0.209	
	P-value^b^	0.975	0.756	
	P-value^c^	0.573	0.206	
**Number of analgesic consumption**	2 weeks	18.18 ± 5.89	15.76 ± 8.03	0.127
	4 weeks	19.29 ± 5.74	21.16 ± 6.47	0.117
	P-value^c^	0.139	**0.003**	

*p*-value^1^: comparison between study groups; P-value^a^: comparison between baseline and 2 weeks; P-value^b^: comparison between baseline and 4 weeks; P-value^c^: comparison between 2 weeks and 4 weeks.

**TABLE 4:** Comparison of WOMAC questionnaire scores (mean ± SD) between basil oil (intervention) and diclofenac gel (control) groups, and also before and after the intervention in each groups.

WOMAC		Basil oil (n = 43)	Diclofenac gel (n = 43)	*p-*value^1^
**Total**	Baseline	40.02 ± 16.22	39.66 ± 17.48	0.898
	2 weeks	32.68 ± 16.78	32.83 ± 18.05	0.970
	4 weeks	29.27 ± 14.48	27.52 ± 16.14	0.354
	P-value^a^	**<0.001**	**<0.001**	
	P-value^b^	**<0.001**	**<0.001**	
	P-value^c^	**0.006**	**<0.001**	
**Pain**	Baseline	8.59 ± 4.13	8.17 ± 4.15	
	2 weeks	6.49 ± 3.71	6.73 ± 4.18	0.642
	4 weeks	5.71 ± 3.21	5.48 ± 3.55	0.887
	P-value^a^	**<0.001**	**<0.001**	0.479
	P-value^b^	**<0.001**	**<0.001**	
	P-value^c^	0.075	0.068	
**Physical Function**	Baseline	29.32 ± 11.39	29.65 ± 12.36	0.898
	2 weeks	24.61 ± 12.22	24.56 ± 13.03	0.744
	4 weeks	22.20 ± 11.00	20.88 ± 12.14	0.239
	P-value^a^	**<0.001**	**<0.001**	
	P-value^b^	**<0.001**	**<0.001**	
	P-value^c^	**0.001**	**<0.001**	
**Stiffness**	Baseline	3.41 ± 2.06	3.02 ± 1.99	0.379
	2 weeks	2.61 ± 2.14	2.65 ± 1.99	0.202
	4 weeks	2.20 ± 1.93	2.12 ± 1.90	0.658
	P-value^a^	**0.001**	**0.034**	
	P-value^b^	**0.008**	**0.002**	
	P-value^c^	0.898	**0.009**	

*p*-value^1^: comparison between study groups. P-value^a^: comparison between baseline and 2 weeks. P-value^b^: comparison between baseline and 4 weeks. P-value^c^: comparison between 2 weeks and 4 weeks.

### 3.4 Tolerability and safety

All patients tolerated basil oil well, and the oil did not induce any adverse event in any of the participants.

## 4 Discussion

To the best of our knowledge, the current study is the first investigation which compared the efficacy of basil oil with diclofenac gel in alleviating knee OA symptoms. Based on the results, no significant differences were observed between the basil oil and diclofenac gel groups in terms of decreasing knee joint pain, assessing patients’ physical function using the WOMAC score and the 8-m walk test, as well as knee flexion angle. However, it is worth noting that significant improvements were observed within each group when analyzing the mentioned variables individually. Therefore, the topical application of basil oil thrice daily over a period of 4 weeks can effectively improve the clinical symptoms of knee OA to the same extent as diclofenac gel, which is considered an efficient method for managing this condition (Mason et al., 2004; Derry et al., 2016). It should be noted that several studies have investigated the efficacy of topical diclofenac in managing the symptoms of osteoarthritis. Previous research has reported pain relief improvement percentages ranging from 36.2% to 44% with the use of topical diclofenac (Zacher et al., 2001; Bookman et al., 2004; Tugwell et al., 2004; Wadsworth et al., 2016). There is a general consensus in the literature that diclofenac is effective in reducing osteoarthritis-related pain and stiffness, as well as enhancing physical function (Bariguian Revel et al., 2020). In our current study, the pain relief improvement percentage with diclofenac gel therapy, as measured on the VAS, was found to be 24.6%.

In general, an effective drug for managing OA should possess analgesic, antioxidant, and anti-inflammatory properties (Bennell et al., 2012; Jabbari et al., 2016; Hashempur et al., 2018). Therefore, we discuss some of the pharmacological activities associated with the treatment of this disease based on recent research.

*O. basilicum,* or basil, is known for its high content of phenolic and flavonoid components, including chlorogenic acid, gallic acid, rutin, quercitrin, quercetin, isoquercitrin, rosmarinic acid, caffeic acid, and kaempferol (Omoba et al., 2019). These natural antioxidant molecules, commonly found in various parts of the herb, have been shown to exhibit excellent free radical scavenging effect *in vitro*, as confirmed by 2,2-Diphenyl-1-picryl hydrazyl radical (DPPH) and hydrogen peroxide assays (Lavanya et al., 2019). Regarding the role of oxidative stress in the senescence of cartilage followed by OA, which includes molecular destruction, apoptosis induction in the chondrocytes, disruption of cartilage matrix homeostasis, impairment of cartilage function, and stimulation of the production of pain mediators, the importance of anti-oxidative agents is increasingly recognized (Yudoh, 2005).

An *in vitro* study was conducted to compare the anti-inflammatory properties of basil methanolic and aqueous extracts on macrophage (RAW264.7), human chondrosarcoma (SW1353), and human primary chondrocytes cell lines. This study demonstrated that the aqueous extract of basil outperforms the methanolic extract in terms of its anti-inflammatory properties. Specifically, the aqueous extract significantly reduced the production of nitric oxide, prostaglandin, total nuclear factor-kappa B, cyclooxygenase-2 protein, and matrix metalloproteinase. Therefore, the aqueous extract of basil has the potential as an effective means of controlling inflammation caused by OA (Raina et al., 2016). Additionally, a molecular study which investigated the anti-inflammatory mechanism of *O. basilicum* demonstrated that the methanolic extract of this medicinal plant extract improved the efficiency of mitochondria and decreased the levels of adipokines in maturing adipocytes, thereby regulating systemic inflammation in macrophages (Subash-Babu et al., 2022).

Another study has confirmed the presence of both peripheral and central anti-nociceptive activities in this herb. The study was carried out on Swiss mice, where an acetic acid-induced writhing test was performed. The essential oil of the herb was subcutaneously injected at doses of 50, 100, and 200 mg/kg. The results indicated that the essential oil was able to reduce the contractions of the abdomen at all doses. Furthermore, at a dose of 200 mg/kg, basil essential oil decreased the paw licking time in the formalin test, while at a dose of 50 mg/kg, it increased latency in the hot-plate test. In thermal stimuli, the analgesic activity of basil essential oil was inhibited by naloxone, which is an opioid antagonist. This suggests that the peripheral anti-nociceptive activity of the herb is associated with the inhibition of pain mediators such as prostaglandins, while the central activity is related to the imitation of the morphine mechanism on opium receptors (Venâncio et al., 2011).

With regards to the inclusion of sesame oil in the traditional basil oil formulation, previous animal studies have highlighted sesame’s anti-inflammatory properties, suggesting its potential in OA treatment. Kong et al. demonstrated sesamin’s inhibition of IL-1β-induced inflammatory responses in chondrocytes, indicating a preventive role in OA progression (Kong et al., 2016). Sesame oil’s effects on oxidative stress and muscle function in OA rats, as shown by Dur-Zong et al., further support its potential in alleviating joint pain (Hsu et al., 2016). A clinical trial on sesame seed supplementation in knee OA patients also reported improved symptoms, aligning with our findings. The use of topical sesame oil in our study aligns with these positive outcomes, reinforcing sesame’s potential effectiveness in OA treatment (Eftekhar Sadat et al., 2013).

In conclusion, preclinical and *in vitro* studies have showcased the anti-inflammatory, analgesic, and antioxidant properties of the components within the traditional basil oil formulation. These findings establish a solid theoretical foundation for the potential therapeutic advantages of basil oil in managing knee osteoarthritis. Moving forward, we acknowledge the importance of conducting further clinical studies to directly assess these specific parameters in patients with knee osteoarthritis to establish a more robust scientific foundation for the observed symptomatic and functional improvements associated with topical basil oil treatment.

In the current study, patients in the diclofenac gel group had greater analgesic consumption at fourth week compared to second week. This phenomenon may be due to possibility of developing a reduced response over time, leading to decreased effectiveness of the medication. Recent research findings suggest that the development of tolerance to the pain-relieving effects of NSAIDs may be influenced by an internal opioid system, potentially involving descending pain modulatory systems. Additionally, individual variations in disease progression and pain tolerance among patients could have influenced the analgesic consumption patterns observed in the diclofenac gel group (Tsiklauri et al., 2018).

### 4.1 Limitations

The current trial had certain methodological limitations. The lack of a placebo control group is indeed a limitation of our study. While our study design focused on comparing the efficacy of topical basil oil to an active control group, we recognize the value of including a placebo group for a more comprehensive assessment of treatment effects. Future studies could benefit from the inclusion of a placebo control group to provide a clearer understanding of the specific benefits attributed to the treatment intervention *versus* the natural course of the disease. Furthermore, the inclusion of sesame oil as the carrier for basil oil raises valid concerns regarding the potential influence of sesame oil on the observed outcomes. Recent research has highlighted the beneficial effects of sesame oil on osteoarthritis, particularly due to the chondroprotective properties of sesamine, the primary phytochemical compound found in sesame (Phitak et al., 2012; Askari et al., 2019). Nevertheless, the primary objective of our trial was to evaluate the efficacy of the traditional basil formulation as prescribed in Persian medicine texts for knee osteoarthritis. Therefore, sesame oil was chosen as the carrier for basil oil in order to maintain the authenticity of the traditional formula being studied.

Another limitation is the age range of the individuals who participated in the investigation as most of them were middle-aged or elderly. Polypharmacy, which is common in this age group, may have affected the results. Ethical concerns prevented us from forbidding the elderly participants from taking their medications, which is another limitation. One more limitation of this study is the potential for unblinding due to the differing consistencies and aromas of the basil oil and diclofenac gel treatments. Despite efforts to maintain blinding with opaque containers and participant instructions, the distinct sensory characteristics of the treatments may have influenced patient perceptions and treatment expectations. Patient expectations are known to impact treatment outcomes, and the potential for unblinding could have introduced bias in the results. While steps were taken to minimize this effect, the inherent differences between the treatments remain a limitation of the study. Furthermore, OA is a chronic disorder, and the short follow-up duration in this trial can be considered a limitation. Long-term medication is typically required for patients with OA, so conducting studies with longer follow-up durations may yield different results regarding efficacy and safety. The gender proportion of participants is also worth noting as a limitation. Approximately 84% of the subjects were female, so the applicability of the results to male patients with knee OA may be restricted.

## 5 Conclusion

In conclusion, the results of this randomized clinical trial suggest that the topical application of basil oil, based on a Persian medicine formula, can improve various signs and symptoms of patients with OA, such as physical function and pain, at a similar level to diclofenac gel. However, conducting trials with larger populations and longer durations is recommended for a more reliable assessment of the efficacy and safety of applying basil oil in clinical practice.

## Data Availability

All data obtained from the trial can be found in the article/Supplementary Material, further information can be obtained by contacting the corresponding author.

## Abbreviations

WOMAC, Western Ontario and McMaster Universities Osteoarthritis Index; OA, Osteoarthritis; NSAIDs, Non-steroidal anti-inflammatory drugs; GC, Gas chromatography; FID, Flame ionization detector; BMI, Body mass index; VAS, Visual Analogue Scale; SD, Standard deviation; GEE, Generalized estimate equation.
