# Erratum: A randomized double-blind active-controlled clinical trial on the efficacy of topical basil (*Ocimum basilicum*) oil in knee osteoarthritis

**DOI:** 10.3389/fphar.2025.1557520

**Published:** 2025-01-27

**Authors:** Frontiers Production Office

**Affiliations:** Frontiers Media SA, Lausanne, Switzerland

**Keywords:** osteoarthritis, herbal medicine, traditional Persian medicine, basil, Ocimum basilicum

Due to a production error, “Alireza Askari” and “Fatemeh Sadat Hasheminasab” were not recognized as co-first authors. The corrected author list with the shared first authorship tag appears below. The publisher apologizes for this mistake.

“Alireza Askari1**†**, Fatemeh Sadat Hasheminasab2,3**†**, Omid Sadeghpour4, Mohammad Mehdi Naghizadehd5, Seyed Ali Ravansalar6, Aida Iraji7 and Mohammad Hashem Hashempur7*.

**†**These authors share first authorship.”

The original version of this article has been updated.

